# Precision Stereotactic Radiotherapy for Spinal Tumors: Mechanism, Efficacy, and Issues

**DOI:** 10.3389/fonc.2020.00826

**Published:** 2020-05-22

**Authors:** Hongqing Zhuang, Hongxia Zhuang, Ning Lang, Jiandong Liu

**Affiliations:** ^1^Department of Radiation Oncology, Peking University Third Hospital, Beijing, China; ^2^Department of Hematology, Weifang People's Hospital, Weifang, China; ^3^Department of Radiology, Peking University Third Hospital, Beijing, China; ^4^Orthopedic Department, No. 971 Hospital of Navy, Qingdao, China

**Keywords:** stereotactic ablative radiotherapy, spinal tumor, efficacy, toxicity, spine

## Abstract

Stereotactic ablative radiotherapy (SABR/SBRT) is a revolutionary technique for tumor therapy. Its advantages are especially beneficial for the treatment spinal tumors. It has a wide range of indications in radiotherapy alone and in preoperative and postoperative treatments for spinal tumor. The mechanism of stereotactic radiotherapy for spinal tumors is special, and completely different from traditional radiotherapy. Compared with traditional radiotherapy, SBRT creates more DNA double-strand breaks, leads to less DNA damage repair, and also has anti-vascular effects, *in situ* vaccine effects and abscopal effect. In the present study, the literature regarding SABR for the treatment of spinal tumors is summarized, and we reviewed characteristics of SABR and spinal tumors, as well as the clinical efficacy and toxicity of SABR in treating spinal tumors. In addition, we proposed several issues around the SABR treatment of spinal tumor, the standard of treatment dose, and the post-treatment follow-up. We also made predictions with respect to future management of spinal tumors, SABR development, multi-modality integration between SABR and other treatments, and other future development trends, thereby providing future research directions as a contribution to the field.

## Introduction

The spine is a common site for primary and metastatic cancers. Especially with the recent advancement in tumor targeting treatments and immunotherapy, spinal metastasis is often discussed, and evaluated in cancer treatment. Treatment for spinal tumors is complicated by the vicinity to the major nerve tracts in the spinal cord. The dose of traditional radiotherapy cannot be increased easily in the spinal cord, making it only a palliative treatment rather than definitive (1, 2). Therefore, improvement in radiotherapy for spinal tumors is critical, and SABR has become an uprising trend in radiotherapy for spinal tumors due to its revolutionary advantages, as discussed below.

### The Mechanism and Unique Characteristics of SABR

#### The Mechanism of SABR

The mechanism of stereotactic radiotherapy for spinal tumors is completely different from traditional radiotherapy. Compared with traditional radiotherapy, SABR creates more double-strand breaks in DNA, results in less DNA damage repair, and even has anti-vascular effects, *in situ* vaccine effects and abscopal effect (3, 4). Therefore, stereotactic radiotherapy is an effective local ablation treatment. In addition, it improves the overall control of the disease through the local control of the disease and through several remote effects (5).

#### The Characteristics of SABR

**Figure 1:**
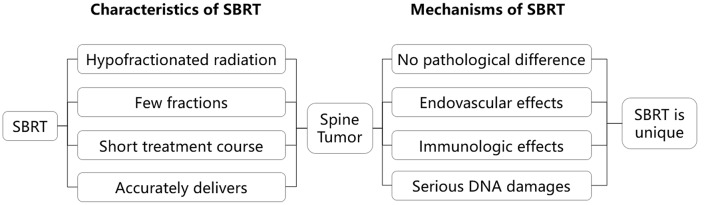
The characteristics of SBRT and its effects on spinal tumor. SBRT is unique. It is “completely different” from traditional fractionated radiation, and SBRT is an ablative treatment.

The advantages of SABR are especially helpful in the treatment of spinal tumors. First, primary and metastatic spinal tumors have a variety of pathologies, with some cell types being more resistant to radiation. SABR, compared to traditional radiotherapy, produces high-dose fractions in a short course of irradiation, making it more effective for radioresistant tumors (6). Second, pain is the most common symptom of patients with spinal tumors, and a short course of irradiation with SABR can relieve pain more quickly. Third, SABR methods can ensure the accuracy of the treatment by tracking movements in between radiation (7–10). However, Traditional radiotherapy cannot easily accommodate for movements during treatment sessions. Fourth, because spinal tumors are often close to the spinal cord, a rapid dose drop outside the target is required. SABR can just achieve a rapid dose drop-off from treatment field to outside of treatment field. Moreover, the treatment days of stereotactic radiotherapy is usually shorter than other radiotherapy methods (for example, IMRT), decreasing cost in staffing and maintenance of hospital facilities. The unique advantages of SABR make it an increasingly popular treatment modality for spinal tumors (Figure 1). It is also important to note that SABR may not be the best option for all patients either. For example, for patients with an expected survival <3 months, 30 Gy in 10 fractions, 20 Gy in 5 fractions, or 8 Gy in 1 fraction with external beam radiotherapy are the reasonable alternative.

### Efficacy of SABR for the Treatment of Spinal Tumors

The drawbacks of traditional radiotherapy make it increasingly incompatible with multimodality treatments for spinal tumors involving new systemic treatments. In comparison, SABR has shown high efficacy and low toxicity for spinal tumors while in conjunction with other treatment modalities. Overall, the application of SABR in spinal tumors include three different ways: primary treatment, repeat treatment after other radiotherapy, and postoperative SABR.

#### SABR as Primary Treatment

SABR as primary treatment is the most important way that SABR is used for spinal tumors, and also the most important research area for SABR in spinal tumors. A representative study of SABR showed that the local control rate of SABR was >80% (11–28), and local control was even higher (>90%) in other studies (29), greatly improved compared with traditional radiotherapy where recurrence rates are close to 80% (30, 31). Moreover, SABR also shows significant benefit in pain relief. The efficacy of SABR promotes the change in radiotherapy for spinal tumor from palliative treatment with traditional radiotherapy to definitive radiation with SABR (32, 33), improving local control and quality of life for patients with spinal tumors (Table 1).

**Table 1:** Selected spine SBRT series for spinal metastases with no prior history of radiation.

**Authors & year**	**Study type**	**No.of Tumors/Patients**	**Histology**	**Total Dose (Range)/No. of Fractions (Range)**	**Follow-up time Months (Range)**	**Local Control**	**Overall Survival**	**Pain Response**
Gerszten et al. (11)	Prospective	156	Mixed	Mean:20 Gy (12.5–25 Gy)/1f	Median: 21 (3–53)	90% (crude)	na	86% reported long-term improvement
Yamada et al. (12)	Retrospective	103/93	Mixed	Median: 24 Gy (18–24 Gy)/1f	Median: 15 (2–45)	90% (15 months)	Median: 15 months	na
Sahgal et al. (13)	Retrospective	23/14	Mixed	Median: 24 Gy (7–40 Gy)/3 (1–5)f	Median: 9 (1–26)	85%(1 year)/69%(2 years)	45% (2 years)	na
Nguyen et al. (14)	Prospective	na/22a	Renal cell carcinoma	Median: 27 Gy (24–30 Gy)/3 (1–5)f	Median: 13.1(3.3–54.5)	82% (1 year)c	72% (1 year)c	BPI:no pain 23%(baseline) to 52% (12 months)
Wang et al. (15)	Prospective	166/149	Mixed	27–30 Gy/3f	Median: 15.9(1.0–91.6)	80.5% (1 year)/72.4%(2 years)	68.5%(1 year)/46.4%(2 years)	BPI:no pain 26% (baseline) to 54% (6 months)
Ahmed et al. (16)	Retrospective	63/46a	Mixed	Median: 24 Gy (10–40 Gy)/3 (1–5)f	Mean: 8.2	91.2% (1 year)	59% (1 year)	na
Thibault et al. (17)	Retrospective	60/37a	Renal cell carcinoma	Median: 24 Gy (18–30 Gy)/2 (1–5)	Median: 12.3(1.2–55.4)	83.4% (1 year)/66.2%(2 years)	64.1%(1 year)/45.6%(2 years)	na
Guckenberger et al. (18)	Retrospective	387/301	Mixed	Median: 24 Gy (10–60 Gy)/3 (1–20)f	Median: 11.8 (0–105)	89.9% (1 year)/83.9%(2 years)	64.9% (1 year)/43.7%(2 years)	na
Sohn et al. (19)	Retrospective	13/13	Renal cell carcinoma	Mean: 38.0 Gy/median: 4f	na	85.7% (1 year)	Median: 15 months	23.1% complete; 53.8% partial
Folkert et al. (20)	Retrospective	108/88a	Sarcoma	Median:24Gy (18–24Gy)/1 or median: 28.5 Gy (24–36 Gy)/3 (3–6)	Median: 12.3(1–80.7)	87.9% (1 year)	60.6% (1 year)	na
Park et al. (21)	Retrospective	45/28a	Mixed	Median: 27 Gy (18–35 Gy)/3 (1–5)f	Median: 7.4(1.1–42.5)	93.2% (1 year)/93.2%(2 years)	47.4% (1 year)/27.9%(2 years)	VAS:median4(pre-SBRT)to 1(1–3 months post-SBRT)
Azad et al. (22)	Retrospective	25/25	Mixed	Median: 20 Gy(15–25.5)/2(1–5)f	Median: 18(1–81)	84.2% (crude)	Median: 28 months	na
Bate et al. (23)	Retrospective	48/36a	Mixed	16–23 Gy/1 or 20–30 Gy/2–5f	Median: 9.8	95.8% (1 year)	44% (crude)	na
Bishop et al. (24)	Retrospective	332/285f	Mixed	Median (tumor dose): 43 Gy	Median: 19(0–111)	88% (1 year)/82% (3 years)	64% (1 year)/33% (3 years)	na
Sellin et al. (25)	Retrospective	40/37	Renal cell carcinoma	Median: 24 Gy (24–30 Gy)/1 (1–5)f	Median: 49.0(38.2–75.8)	57%	Median: 16.3 months	VAS: 41.4% improved pain
Anand et al. (26)	Retrospective	76/52e	Mixed	Median: 24 Gy (24–27 Gy)/3 (1–3)f	Median: 8.5(3.0–40.0)	94% (1 year)/82.6%(2 years)	68% (1 year)/45.4%(2 years)	92.3% complete; 5.8% partial
Ghia et al. (28)	Prospective	28/28	Mixed	18 or 24 Gy/1f	Median:17 (12.7–21.0)	89% (1 year)	Median: 28.6 months	na
Tseng et al. (27)	Prospective	279/145	Mixed	24Gy/2f	Median:15 (0.1–71.6)	1-year local failure: 9.7%	1-year OS:73.1%	na

#### SABR Treatment After Prior Radiotherapy

Recurrence after prior radiotherapy is common in the treatment of spinal tumor. Due to the dose limitation on spinal cord, ordinary radiotherapy cannot be repeated at sites that received prior radiotherapy. Therefore, SABR is the only option for repeat radiation. The results (13, 17, 34–39) demonstrated that repeat SABR achieved good efficacy in controlling tumor-related pain (Table 2).

**Table 2:** Selected re-irradiation spine SABR series for spinal metastases.

**Authors & Year**	**Study type**	**No. of Tumors/No.of Patients**	**Histology**	**Prior RT Dose (Range)/No. of Fractions (Range)**	**Total Dose(Range)/No. of Fractions (Range)**	**Follow-up in Months(Range)**	**Local Control**	**Overall Survival**	**Pain Response**
Sahgal et al. (13)	Retrospective	37/25	Mixed	Median: 24 Gy (7–40 Gy)/3 (1–5)	Median: 36 Gy/14	Median: 7 (1–48)	92% (1 year)	45% (2 years)^a^	na
Mahadevan et al. (38)	Retrospective	81/60	Mixed	Median: 24 Gy (24–30 Gy)/3 (3–5)	Median: 30 Gy (8–46 Gy)/10 (1–25)	Median: 12 (4–36)	Median: 9 months	Median: 11 months	4.7% reported pain response; 18% complete response
Choi et al. (35)	Retrospective	51/42	Mixed	Median: 20 Gy (10–30 Gy)/2 (1–5)	Median: 40 Gy (30–40 Gy)/20 (10–20)	Median: 7 (2–47)	73% (1 year)	68% (1 year)	65% reported pain response
Garg et al. (34)	Prospective	63/59	Mixed	Median: 27 Gy (20–30 Gy)/3 (3–5)	Median: 30 Gy/na	Median: 13 (0.9–67.5)	76% (1 year)	76% (1 year)	na
Damast et al. (36)	Retrospective	97/95	Mixed	Median: 30 Gy (16–30 Gy)/5 (4–6)	Median: 30 Gy (8–66 Gy)/na	Median: 12.1 (0.2–63.6)	66% (1 year)	52–59% (1 year); median: 13.6 months	77% reported pain response
Thibault et al. (17)	Retrospective	11/37	Renal cell carcinoma	Median: 24 Gy (18–30 Gy)/2 (1–5)	Median: 30 Gy (8–30 Gy)/10 (1–10)	Median: 12.3 (1.2–55.4)	83.4% (1 year)/66.2% (2 years)	64.1% (1 year)/45.6% (2 years)	na
Thibault et al. (39)	Retrospective	56/40	Mixed	Median: 30 Gy (20–35 Gy)/4 (2–5)	Median (SBRT): 24 Gy(20–35 Gy)/2(1–5); median (cEBRT, n $\frac{1}{4}$ 24):22.5 Gy (20–30 Gy)/5 (5–40)	Median: 6.8 (0.9–39)	80.6% (1 year)/71.5% (2 years)	48% (1 year)	na
Kawashiro et al. (37)	Retrospective	23/23	Mixed	Median: 24.5 Gy (14.7–50 Gy)/5 (3–25)	Median: 30 Gy (30–40 Gy)/10 (10–20)	Median: 10 (1–54)	88% (1 year)/75% (2 years)	50% (1 year)/20% (2 years)	78.9% reported pain relief

##### Postoperative SABR

The role of decompressive surgery in patients with symptomatic single-level MESCC was established by Patchell et al. (40). This article demonstrated the effect of surgery and postoperative radiation as a standard in the treatment of MESCC. Moreover, several studies of stereotactic radiotherapy have also confirmed that stereotactic radiotherapy has better advantages for postoperative treatment of spinal tumors. Stereotactic radiotherapy can achieve better local control (Based on the available data, the rate of local control is about 80–90%) and pain relief (17, 22, 41–50), although treatment dose and fraction greatly varied in the published series (Table 3).

**Table 3:** Selected postoperative spine SABR series for spinal metastases.

**Study authors (Year)**	**Study design**	**No. of Tumors/No.of Patients**	**Histology**	**Total dose (Range)/No. of Fractions (Range)**	**Follow-up in Months(Range)**	**Local Control**	**Overall survival**	**Pain response**
Gerszten et al. (41)	Prospective	26/26	Mixed	Mean: 18 Gy to 80% isodose line (16–20 Gy)/1	Median: 16 (11–24)	na	na	VAS: 92% long-term improvement
Rock et al. (42)	Retrospective	18/18	Mixed	Mean: 11.4 Gy (6–16)/1	Median: 7 (4–36)	na	na	na
Gerszten et al. (43)	Prospective	11/11	Mixed	Mean: 19 Gy (16–22.5 Gy)/1	Median: 11 (7–44)	na	na	VAS: 100% long-term improvement
Moulding et al. (44)	Retrospective	21/21	Mixed	Median: 24 Gy (18–24 Gy)/1	Median: 10.2 (1.2–54.0)	90.5% (1 year)	Median: 10.2 months	na
Massicotte et al. (45)	Retrospective	10/10	Mixed	Median: 24 Gy (18–35 Gy)/3 (1–5)	Median: 13 (3–18)	70% (crude)	na	na
Al-Omair et al. (46)	Retrospective	80/80	Mixed	Median: 24 Gy (18–40 Gy)/2 (1–5)	Median: 8.3 (0.13–39.1)	84% (1 year)	64% (1 year)	na
Laufer et al. (47)	Retrospective	186/186	Mixed	24 Gy/1 (21.5%) or 24–30 Gy/3 (19.9%), or 18–36 Gy/5–6 (58.6%)	Median: 7.6 (1.0–66.4)	83.6% (1 year)	29.0% (crude); median among patients who died: 6.1 months	na
Azad et al. (22)	Retrospective	21/21	Mixed	16–22 Gy/1 or 20–30 Gy/2–5	Median: 13.7	90.5% (1 year)	44%^a^ (crude)	na
Zabi Wardak et al. (48)	Prospective	29/25	Mixed	20 Gy/1	Median: 9.6	92%	na	VAS: 91% significantly improved
Redmond et al. (49)	Prospective	33/35	Mixed	30 Gy/5f	na	90% (1 year)	na	na

In conclusion, SABR has shown great efficacy in treating spinal tumors as a primary treatment, as salvage treatment after prior radiotherapy and as postoperative radiotherapy. Compared to traditional radiotherapy, SABR for spinal tumors is more effective in symptom relief, tumor control, and potentially improves survival.

### Practical Questions When Using SABR for the Treatment of Spinal Tumors

#### Selection of Equipment of SABR: What Are the Differences Between Different Devices?

SABR could be used with different treatment platforms from different companies. The similarities and differences of these devices are also frequently asked by patients in clinical practice. The accuracy, efficacy, and cost-efficiency of equipment are the main factors for hospitals to decide on equipment. Among all current treatment platforms, some devices use CBCT as the treatment accuracy support equipment, the Cyberknife has a real-time tracking system. This is the advantage of CyberKnife, but there are also a lot of disadvantages, such as no posterior beams, more anterior spillage in the visceral organs as all beam come from the front and side. Further, CyberKnife treatment has long delivery time. There are many top centers using LINAC-based SABR for spine tumors in the world. Cost-efficiency is another crucial criterion for the assessment of the treatment equipment. For example, the cost of Cyberknife treatments is high in china and some countries. Therefore, selecting the appropriate equipment and treatment is an important consideration when SABR for spinal tumors, but different devices have different advantages and disadvantages (27, 51–54). The ideal equipment for clinical needs is a radiotherapy machine with real-time tracking system, full angle radiation field and short time to complete treatment. However, doctors' professional experience, academic level and a good teamwork maybe more important factors than equipment.

#### Understanding the Balance Between Tumor Control and Radiation-Related Adverse Events in the Treatment Process: Deciding Treatment Dosage

The goal of tumor treatment is to control the tumor and reduce injury to surround tissue. However, in many cases, the tumor cannot be controlled without damaging surrounding tissue. If left uncontrolled, spinal tumors often inevitably lead to spinal cord injury. Therefore, the benefits of radiotherapy for spinal tumors still outweighs its harm. The current standard doses used in radiation for spinal tumors are usually low enough to avoid damaging neurologic structures in the spinal cord (22, 23). Clinical practice in choosing dosage for spinal cord irradiation can be mainly divided into two situations. First, in the case of achieving spinal tumor control without damaging the spinal cord, it is necessary to achieve the two goals at the same time. Second, in the case of tumor control where uncontrolled tumor growth causes spinal cord injury, an optimal dose to control the tumor is critical and the first priority. This phenomenon indicated that SABR dosing selection is the key to improve spinal tumor treatment and requires further research.

#### Efficacy Evaluation After SABR for Spinal Tumors: How to Study the Efficacy of SABR for Spinal Tumors?

Spinal tumor is different from other solid tumors of other organs. Radiologic changes are sometimes not the best representation of tumor control after radiation treatment. Therefore, the commonly used criterion RECIST does not apply to the evaluation after tumor control after radiotherapy of spinal tumors (55). The spine response assessment In Neuro-Oncology (SPINO) group present the first report on the challenges in standardizing imaging-based assessment of local control and pain for spinal metastases. The ultimate goal of the SPINO group is to report consensus criteria for tumor imaging, clinical assessment, and symptom-based response criteria to help standardize the evaluation (56). The SPINO standard improved the evaluation of spinal tumors after fusion of different clinical factors. However, there are still many clinical puzzles in clinical practice. After all, the evaluation of spinal tumors after radiotherapy is very complicated. Overall, the combination of radiologic changes in the setting of comprehensive consideration for metabolic and functional changes is likely the future direction for evaluating spinal cord tumor control after radiotherapy. First, evaluating tumor control after SABR for spinal tumors requires a combination of multiple radiology modalities: CT is used to observe the bone mass, MRI for morphology, and ECT and PET for metabolic activity. Second, imaging techniques such as functional nuclear magnetics and other new evaluation methods (for example: artificial intelligence) of spinal tumors are being developed, which may play a potentialrole in predicting the prognosis on spinal tumor and in evaluating treatment response after SABR. In conclusion, evaluating treatment response of spinal tumors after SABR is an area for further investigation, with the integration of radiological, functional, and metabolic changes as a novel direction for studying the efficacy of SABR.

In conclusion, as a revolutionary technique for tumor treatment, SABR has several advantages that makes it a good treatment modality for spinal tumors. As a result, SABR has shown excellent efficacy as primary treatment, repeat radiation treatment, and postoperative radiotherapy for spinal tumors. Spinal tumor is one of the best indications for SABR, and SABR is becoming part of the backbone of spinal tumor treatment. With several issues remain regarding the selection of specific equipment and type of SABR, standardization of radiation dose, and evaluation of treatment response, more will come in the future with the development of SABR, further accumulation of clinical data, and integration of SABR into multi-disciplinary cancer treatment.

## Author Contributions

HongqZ and JL participated in the idea of the article. HongqZ and NL collected the data. HongqZ and HongxZ wrote the paper. All authors were responsible for the final review of the manuscript.

## Conflict of Interest

The authors declare that the research was conducted in the absence of any commercial or financial relationships that could be construed as a potential conflict of interest.
